# Bis(μ-2,4-dihy­droxy­benzoato-κ^2^ *O*:*O*′)bis­[aqua­(2,4-dihy­droxy­benzoato-κ*O*)(1,10-phenanthroline-κ^2^ *N*,*N*′)cadmium(II)]

**DOI:** 10.1107/S1600536810021252

**Published:** 2010-06-09

**Authors:** Jing-Jing Nie, Tian-Tian Pan, Jian-Rong Su, Duan-Jun Xu

**Affiliations:** a Department of Chemistry, Zhejiang University, People’s Republic of China

## Abstract

In the title centrosymmetric dimeric Cd^II^ complex, [Cd_2_(C_7_H_5_O_4_)_4_(C_12_H_8_N_2_)_2_(H_2_O)_2_], the Cd^II^ cation is coord­inated by a bidentate phenanthroline (phen) ligand, three dihy­droxy­benzoate (dhba) anions and one water mol­ecule in a distorted CdN_2_O_4_ octa­hedral geometry. Among the dhba anions, two anions bridge two Cd^II^ cations to form the dimeric complex with significant different Cd—O bond distances of 2.2215 (19) and 2.406 (2) Å. The centroid–centroid distance of 3.4615 (19) Å between two nearly parallel benzene rings of the dhba and phen ligands coordinating to the same Cd^II^ cation indicates the existence of intra­molecular π–π stacking in the complex. Extensive O—H⋯O hydrogen bonding and inter­molecular weak C—H⋯O hydrogen bonding help to stabilize the crystal structure. One hy­droxy group of the monodentate dhba ligand is disordered over two sites with a site-occupancy ratio of 0.9:0.1.

## Related literature

For the correlation between π–π stacking and electron-transfer processes in some biological systems, see: Deisenhofer & Michel (1989 ▶). For general background to π–π stacking, see: Li *et al.* (2005 ▶). For π–π stacking involving a dihy­droxy­benzoate ligand in an Ni complex, see: Yang *et al.* (2006 ▶). Intramol­ecular π–π stacking was previously observed in a Sr complex with a hy­droxy­benzoate ligand, see: Su *et al.* (2005 ▶).
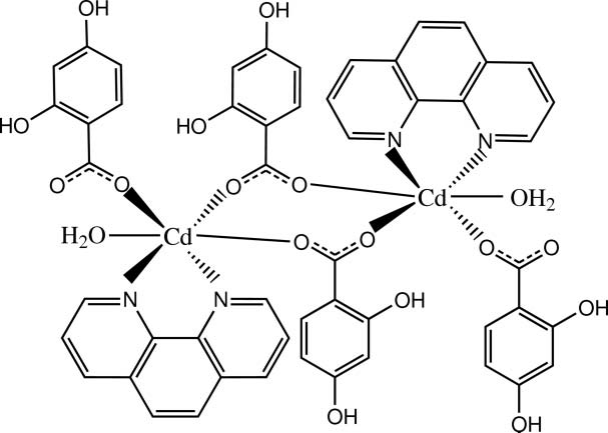


## Experimental

### 

#### Crystal data

[Cd_2_(C_7_H_5_O_4_)_4_(C_12_H_8_N_2_)_2_(H_2_O)_2_]
*M* *_r_* = 1233.68
Monoclinic, 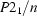

*a* = 14.899 (4) Å
*b* = 6.874 (2) Å
*c* = 23.400 (6) Å
β = 103.378 (2)°
*V* = 2331.4 (11) Å^3^
*Z* = 2
Mo *K*α radiation
μ = 1.00 mm^−1^
*T* = 296 K
0.26 × 0.17 × 0.11 mm

#### Data collection

Rigaku R-AXIS RAPID diffractometer
Absorption correction: multi-scan (*ABSCOR*; Higashi, 1995 ▶) *T*_min_ = 0.758, *T*_max_ = 0.890
19273 measured reflections
4654 independent reflections
4015 reflections with *I* > 2σ(*I*)
*R*_int_ = 0.031

#### Refinement

*R*[*F* ^2^ > 2σ(*F* ^2^)] = 0.028
*wR*(*F* ^2^) = 0.067
*S* = 1.11
4654 reflections
358 parameters
3 restraints
H atoms treated by a mixture of independent and constrained refinement
Δρ_max_ = 0.66 e Å^−3^
Δρ_min_ = −0.38 e Å^−3^

### 

Data collection: *PROCESS-AUTO* (Rigaku, 1998 ▶); cell refinement: *PROCESS-AUTO*; data reduction: *CrystalStructure* (Rigaku/MSC, 2002 ▶); program(s) used to solve structure: *SIR92* (Altomare *et al.*, 1993 ▶); program(s) used to refine structure: *SHELXL97* (Sheldrick, 2008 ▶); molecular graphics: *ORTEP-3 for Windows* (Farrugia, 1997 ▶); software used to prepare material for publication: *WinGX* (Farrugia, 1999 ▶).

## Supplementary Material

Crystal structure: contains datablocks I, global. DOI: 10.1107/S1600536810021252/ng2775sup1.cif

Structure factors: contains datablocks I. DOI: 10.1107/S1600536810021252/ng2775Isup2.hkl

Additional supplementary materials:  crystallographic information; 3D view; checkCIF report

## Figures and Tables

**Table 1:** Selected bond lengths (Å)

Cd—N1	2.326 (2)
Cd—N2	2.324 (2)
Cd—O1	2.215 (2)
Cd—O5	2.406 (2)
Cd—O6^i^	2.2215 (19)
Cd—O9	2.361 (2)

Symmetry code: (i) 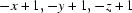.

**Table 2:** Hydrogen-bond geometry (Å, °)

*D*—H⋯*A*	*D*—H	H⋯*A*	*D*⋯*A*	*D*—H⋯*A*
O3—H3*A*⋯O2	0.82	1.81	2.540 (3)	148
O3′—H3*B*⋯O1	0.82	1.56	2.332 (19)	156
O4—H4*A*⋯O7^ii^	0.82	1.91	2.719 (3)	172
O7—H7*A*⋯O6	0.82	1.80	2.523 (3)	147
O8—H8*A*⋯O3^iii^	0.82	1.94	2.752 (3)	169
O9—H9*A*⋯O2	0.86 (1)	1.97 (2)	2.738 (3)	148 (3)
O9—H9*B*⋯O5^iv^	0.86 (1)	2.03 (2)	2.842 (3)	158 (3)
C24—H24⋯O5^i^	0.93	2.58	3.433 (4)	152

Symmetry codes: (i) 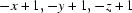; (ii) 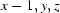; (iii) 
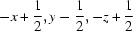
; (iv) 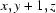.

**Fig. 1.**
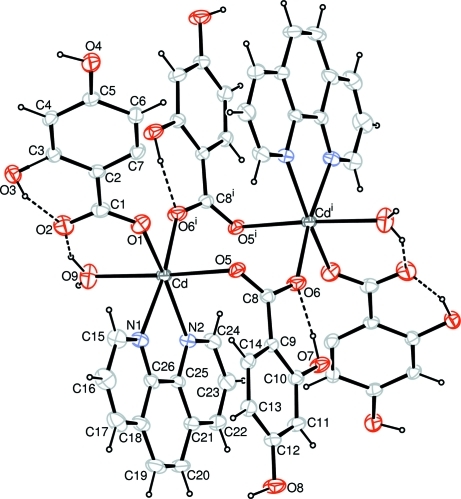
The dimeric molecular structure of the title complex with 30% probability displacement ellipsoids (arbitrary spheres for H atoms). The minor dosordered component is omitted for clarity. Dashed lines indicate intramolecular hydrogen bonding [symmetry code: (i) 1 - x,1 - y,1 - z].

**Fig. 2.**
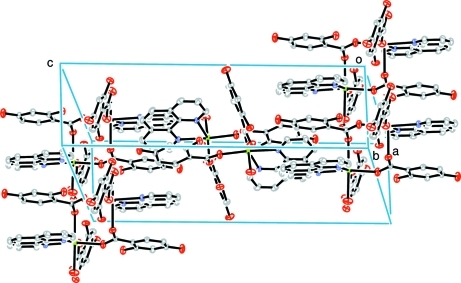
The unit cell packing showing intermolecular π-π stacking between dhba and phen rings.

## supplementary crystallographic information

### Comment

The π-π stacking between aromatic rings is an important non-covalent interaction and correlated with the electron transfer process in some biological systems (Deisenhofer & Michel, 1989). As a continuous work of the series investigation on the nature of π-π stacking (Li *et al.*, 2005), we prepared the title Cd^II^ complex which contains a double-hydroxy substituted benzoate ligand. We present here its crystal structure to compare with those incorporating monohydroxybenzoate ligand and to show the effect of hydroxy substitution on the benzene ring to π-π stacking between aromatic rings.

The molecular structure of the title compound is shown in Fig. 1. The dimeric Cd^II^ complex locates across on an inversion center. Each Cd^II^ atom is coordinated by one phenanthroline (phen), one water molecule and three dihydroxybenzoate (dhba) dianions in a distorted octahedral geometry (Table 1). The C1-containg dhba is monodentately coordinated to a Cd^II^ atom and is well coplanar with the Cd atom [the maximum atomic deviation 0.071 (3) Å (Cd)] while the C8-conating dhba bridges two Cd atoms to form the dimeric complex.

It is notable that intra-molecular π-π stacking exists in the dimeric complex. Partially overlapped arrangement between nearly parallel C9-containg benzene ring and phen ring system [dihedral angle 6.56°] is observed in the molecular structure; the shorter centroids distance of 3.4615 (19) Å between C9-benzene and C19-benzene rings suggests the existence of intramolecualr π-π stacking. The perpendicular distance of centroid of C9-benzene ring on C19-benzene ring and perpendicular distance of centroid of C19-benzene ring on C9-benzene ring are 3.365 and 3.388 Å. Intra-molecular π-π stacking was previously observed in a Sr^II^ complex with hydroxybenzoate ligand (Su *et al.*, 2007). π-π stacking involving dhba ligand in a Ni^II^ complex was reported by Yang *et al.* (2006).

Intermolecular π-π stacking is also present in the crystal structure; the centroid distance between C9-ring and C19^i^-ring is 3.754 (2) Å [symmetry code: (i) *x*, -1 + *y*, *z*] (Fig. 2).

Extensive O—H···O hydrogen bonding and intermolecular weak C—H···O hydrogen bonding (Table 2) help to stabilize the crystal structure.

### Experimental

CdCl_2_^.^2.5H_2_O (0.46 g, 2 mmol), Na_2_CO_3_ (0.21 g, 2 mmol) and 2,4-dihydroxybenzoic acid (0.31 g, 2 mmol) was dissolved in a water/ethanol solution (20 ml, 1:3); then phenanthroline (0.36 g, 2 mmol) was added to above solution. The mixture was refluxed for 3 h, and then filtered after cooling to room temperature. Colorless single crystals of the title compound were obtained after one week.

### Refinement

One hydroxy group of C1-containing dhba ligand is disordered, site occupancies for O3 and O3' atoms were initially refined and converged to 0.895 (3) and 0.105 (3), they were fixed as 0.9 and 0.1 respectively in the final refinement. Water H atoms were located in a difference Fourier map and refined with O—H distance restrains, *U*_iso_(H) = 1.2*U*_eq_(O). Other H atoms were placed in calculated positions with O—H = 0.82 and C—H = 0.93 Å, and refined in riding mode with *U*_iso_(H) = 1.2*U*_eq_(O,*C*). In the final refinement the C6—C7 distance was constrained to be 1.38±0.01 Å.

### Crystal data

**Table:**

[Cd_2_(C_7_H_5_O_4_)_4_(C_12_H_8_N_2_)_2_(H_2_O)_2_]	*F*(000) = 1240
*M**_r_* = 1233.68	*D*_x_ = 1.757 Mg m^−^^3^
Monoclinic, *P*2_1_/*n*	Mo *K*α radiation, λ = 0.71073 Å
Hall symbol: -P 2yn	Cell parameters from 15860 reflections
*a* = 14.899 (4) Å	θ = 3.1–26.0°
*b* = 6.874 (2) Å	µ = 1.00 mm^−^^1^
*c* = 23.400 (6) Å	*T* = 296 K
β = 103.378 (2)°	Prism, colorless
*V* = 2331.4 (11) Å^3^	0.26 × 0.17 × 0.11 mm
*Z* = 2	

### Data collection

**Table:**

Rigaku R-AXIS RAPID diffractometer	4654 independent reflections
Radiation source: fine-focus sealed tube	4015 reflections with *I* > 2σ(*I*)
graphite	*R*_int_ = 0.031
Detector resolution: 10.0 pixels mm^-1^	θ_max_ = 26.1°, θ_min_ = 3.1°
ω scans	*h* = −18→18
Absorption correction: multi-scan (*ABSCOR*; Higashi, 1995)	*k* = −8→8
*T*_min_ = 0.758, *T*_max_ = 0.890	*l* = −29→29
19273 measured reflections	

### Refinement

**Table:**

Refinement on *F*^2^	Primary atom site location: structure-invariant direct methods
Least-squares matrix: full	Secondary atom site location: difference Fourier map
*R*[*F*^2^ > 2σ(*F*^2^)] = 0.028	Hydrogen site location: inferred from neighbouring sites
*wR*(*F*^2^) = 0.067	H atoms treated by a mixture of independent and constrained refinement
*S* = 1.11	*w* = 1/[σ^2^(*F*_o_^2^) + (0.0281*P*)^2^ + 1.5199*P*] where *P* = (*F*_o_^2^ + 2*F*_c_^2^)/3
4654 reflections	(Δ/σ)_max_ = 0.001
358 parameters	Δρ_max_ = 0.66 e Å^−^^3^
3 restraints	Δρ_min_ = −0.38 e Å^−^^3^

### Special details

**Table:**

Geometry. All e.s.d.'s (except the e.s.d. in the dihedral angle between two l.s. planes) are estimated using the full covariance matrix. The cell e.s.d.'s are taken into account individually in the estimation of e.s.d.'s in distances, angles and torsion angles; correlations between e.s.d.'s in cell parameters are only used when they are defined by crystal symmetry. An approximate (isotropic) treatment of cell e.s.d.'s is used for estimating e.s.d.'s involving l.s. planes.
Refinement. Refinement of *F*^2^ against ALL reflections. The weighted *R*-factor *wR* and goodness of fit *S* are based on *F*^2^, conventional *R*-factors *R* are based on *F*, with *F* set to zero for negative *F*^2^. The threshold expression of *F*^2^ > σ(*F*^2^) is used only for calculating *R*-factors(gt) *etc*. and is not relevant to the choice of reflections for refinement. *R*-factors based on *F*^2^ are statistically about twice as large as those based on *F*, and *R*- factors based on ALL data will be even larger.

### Fractional atomic coordinates and isotropic or equivalent isotropic displacement parameters (Å^2^)

**Table:**

	*x*	*y*	*z*	*U*_iso_*/*U*_eq_	Occ. (<1)
Cd	0.397190 (12)	0.67327 (3)	0.425111 (8)	0.03048 (7)	
N1	0.37208 (16)	0.6779 (3)	0.32325 (9)	0.0369 (5)	
N2	0.53505 (15)	0.7623 (3)	0.40192 (9)	0.0329 (5)	
O1	0.25877 (13)	0.5555 (3)	0.42507 (10)	0.0534 (6)	
O2	0.16636 (15)	0.8085 (3)	0.39648 (12)	0.0592 (6)	
O3	−0.00646 (15)	0.7698 (3)	0.38480 (10)	0.0421 (5)	0.90
H3A	0.0419	0.8218	0.3821	0.063*	0.90
O3'	0.2006 (13)	0.254 (3)	0.4445 (9)	0.040 (4)	0.10
H3B	0.2343	0.3426	0.4388	0.060*	0.10
O4	−0.11493 (14)	0.1736 (3)	0.44782 (11)	0.0531 (5)	
H4A	−0.1643	0.2313	0.4387	0.080*	
O5	0.44142 (12)	0.3360 (3)	0.43181 (8)	0.0376 (4)	
O6	0.58823 (14)	0.2947 (3)	0.47871 (8)	0.0465 (5)	
O7	0.71417 (12)	0.3368 (3)	0.42386 (8)	0.0453 (5)	
H7A	0.6910	0.3342	0.4524	0.068*	
O8	0.63878 (16)	0.2938 (4)	0.21875 (9)	0.0554 (6)	
H8A	0.5953	0.2774	0.1905	0.083*	
O9	0.33412 (15)	0.9899 (3)	0.41432 (12)	0.0584 (6)	
H9A	0.2763 (6)	0.977 (6)	0.4129 (16)	0.070*	
H9B	0.353 (3)	1.1085 (18)	0.4142 (16)	0.070*	
C1	0.18095 (18)	0.6359 (5)	0.41415 (12)	0.0408 (7)	
C2	0.10138 (17)	0.5164 (4)	0.42243 (10)	0.0324 (5)	
C3	0.01181 (18)	0.5888 (4)	0.40876 (11)	0.0330 (6)	
H3	0.0011	0.7142	0.3939	0.040*	0.10
C4	−0.06174 (17)	0.4778 (4)	0.41683 (11)	0.0350 (6)	
H4	−0.1212	0.5289	0.4077	0.042*	
C5	−0.04658 (18)	0.2906 (4)	0.43854 (12)	0.0349 (6)	
C6	0.04272 (16)	0.2132 (4)	0.45253 (13)	0.0386 (6)	
H6	0.0532	0.0874	0.4672	0.046*	
C7	0.11485 (15)	0.3269 (4)	0.44412 (11)	0.0366 (6)	
H7	0.1742	0.2758	0.4532	0.044*	0.90
C8	0.52452 (18)	0.3071 (4)	0.43170 (11)	0.0314 (5)	
C9	0.55409 (17)	0.2915 (3)	0.37581 (11)	0.0295 (5)	
C10	0.64719 (18)	0.3133 (4)	0.37392 (11)	0.0338 (5)	
C11	0.67359 (19)	0.3138 (4)	0.32113 (12)	0.0412 (6)	
H11	0.7354	0.3295	0.3206	0.049*	
C12	0.6079 (2)	0.2908 (4)	0.26885 (12)	0.0384 (6)	
C13	0.5157 (2)	0.2642 (4)	0.26956 (12)	0.0378 (6)	
H13	0.4717	0.2452	0.2347	0.045*	
C14	0.49017 (18)	0.2662 (4)	0.32251 (12)	0.0343 (6)	
H14	0.4283	0.2501	0.3227	0.041*	
C15	0.2934 (2)	0.6307 (5)	0.28578 (13)	0.0469 (7)	
H15	0.2427	0.5985	0.3007	0.056*	
C16	0.2839 (3)	0.6274 (5)	0.22552 (14)	0.0568 (9)	
H16	0.2281	0.5924	0.2006	0.068*	
C17	0.3582 (3)	0.6768 (5)	0.20315 (13)	0.0541 (8)	
H17	0.3529	0.6768	0.1628	0.065*	
C18	0.4424 (2)	0.7273 (4)	0.24132 (12)	0.0425 (7)	
C19	0.5236 (3)	0.7702 (5)	0.22142 (14)	0.0523 (8)	
H19	0.5212	0.7717	0.1813	0.063*	
C20	0.6036 (3)	0.8083 (5)	0.25983 (15)	0.0525 (8)	
H20	0.6557	0.8362	0.2458	0.063*	
C21	0.6106 (2)	0.8070 (4)	0.32217 (13)	0.0404 (6)	
C22	0.6939 (2)	0.8440 (5)	0.36345 (15)	0.0512 (8)	
H22	0.7474	0.8713	0.3509	0.061*	
C23	0.6955 (2)	0.8396 (5)	0.42162 (15)	0.0502 (8)	
H23	0.7498	0.8653	0.4493	0.060*	
C24	0.6148 (2)	0.7962 (4)	0.43937 (13)	0.0420 (7)	
H24	0.6170	0.7908	0.4794	0.050*	
C25	0.53247 (19)	0.7677 (4)	0.34348 (11)	0.0325 (6)	
C26	0.4460 (2)	0.7251 (4)	0.30205 (11)	0.0337 (6)	

### Atomic displacement parameters (Å^2^)

**Table:**

	*U*^11^	*U*^22^	*U*^33^	*U*^12^	*U*^13^	*U*^23^
Cd	0.02787 (10)	0.03859 (11)	0.02644 (10)	−0.00149 (8)	0.00929 (7)	0.00251 (8)
N1	0.0418 (12)	0.0401 (12)	0.0289 (11)	−0.0037 (11)	0.0084 (9)	−0.0006 (10)
N2	0.0353 (12)	0.0348 (11)	0.0296 (11)	−0.0047 (9)	0.0100 (9)	0.0011 (9)
O1	0.0253 (10)	0.0670 (14)	0.0680 (14)	−0.0048 (10)	0.0112 (9)	0.0102 (12)
O2	0.0403 (12)	0.0583 (14)	0.0801 (16)	−0.0100 (10)	0.0161 (11)	0.0206 (13)
O3	0.0364 (12)	0.0399 (12)	0.0486 (13)	−0.0017 (9)	0.0067 (10)	0.0083 (10)
O3'	0.029 (9)	0.031 (9)	0.064 (13)	0.004 (8)	0.016 (9)	0.006 (9)
O4	0.0311 (10)	0.0518 (12)	0.0791 (15)	−0.0038 (9)	0.0182 (10)	0.0162 (12)
O5	0.0328 (10)	0.0374 (10)	0.0479 (11)	−0.0010 (8)	0.0202 (8)	0.0007 (9)
O6	0.0390 (11)	0.0724 (15)	0.0296 (10)	0.0036 (10)	0.0107 (8)	−0.0020 (10)
O7	0.0266 (9)	0.0754 (14)	0.0338 (10)	−0.0018 (10)	0.0067 (8)	−0.0044 (10)
O8	0.0514 (13)	0.0872 (17)	0.0321 (10)	−0.0028 (12)	0.0187 (9)	−0.0037 (11)
O9	0.0403 (12)	0.0381 (11)	0.0909 (17)	−0.0028 (10)	0.0029 (12)	0.0015 (12)
C1	0.0298 (14)	0.0571 (19)	0.0354 (14)	−0.0078 (13)	0.0073 (11)	0.0004 (13)
C2	0.0264 (12)	0.0460 (15)	0.0249 (11)	−0.0032 (11)	0.0062 (10)	−0.0005 (11)
C3	0.0326 (13)	0.0366 (13)	0.0290 (12)	0.0004 (11)	0.0054 (10)	0.0004 (11)
C4	0.0230 (12)	0.0472 (16)	0.0351 (13)	0.0031 (11)	0.0077 (10)	0.0008 (12)
C5	0.0297 (13)	0.0415 (15)	0.0352 (13)	−0.0050 (11)	0.0112 (11)	−0.0012 (12)
C6	0.0342 (14)	0.0377 (16)	0.0445 (15)	0.0029 (11)	0.0103 (12)	0.0067 (12)
C7	0.0238 (12)	0.0508 (16)	0.0348 (13)	0.0049 (12)	0.0057 (10)	0.0010 (13)
C8	0.0352 (14)	0.0268 (12)	0.0344 (13)	−0.0023 (11)	0.0127 (11)	0.0006 (10)
C9	0.0302 (13)	0.0291 (13)	0.0303 (12)	0.0017 (10)	0.0095 (10)	0.0008 (10)
C10	0.0299 (13)	0.0385 (14)	0.0329 (13)	−0.0012 (11)	0.0073 (10)	0.0013 (11)
C11	0.0332 (14)	0.0554 (17)	0.0382 (14)	−0.0019 (13)	0.0151 (12)	−0.0014 (13)
C12	0.0438 (16)	0.0425 (16)	0.0328 (14)	0.0027 (12)	0.0166 (12)	0.0010 (12)
C13	0.0397 (15)	0.0419 (15)	0.0291 (13)	0.0041 (12)	0.0026 (11)	−0.0043 (12)
C14	0.0290 (13)	0.0365 (14)	0.0367 (14)	0.0006 (11)	0.0061 (11)	−0.0024 (11)
C15	0.0464 (17)	0.0512 (18)	0.0398 (15)	−0.0067 (14)	0.0027 (13)	−0.0059 (14)
C16	0.061 (2)	0.063 (2)	0.0383 (16)	−0.0058 (17)	−0.0045 (15)	−0.0064 (15)
C17	0.081 (2)	0.0488 (17)	0.0279 (14)	0.0025 (17)	0.0036 (15)	−0.0025 (13)
C18	0.069 (2)	0.0309 (14)	0.0298 (14)	0.0020 (13)	0.0174 (14)	0.0001 (11)
C19	0.083 (3)	0.0474 (18)	0.0335 (15)	0.0018 (17)	0.0284 (16)	0.0039 (13)
C20	0.068 (2)	0.0501 (18)	0.0532 (19)	0.0054 (16)	0.0422 (18)	0.0091 (15)
C21	0.0474 (16)	0.0354 (14)	0.0449 (16)	0.0050 (13)	0.0239 (13)	0.0077 (12)
C22	0.0439 (17)	0.0514 (18)	0.065 (2)	0.0025 (14)	0.0273 (15)	0.0150 (16)
C23	0.0346 (15)	0.0570 (19)	0.0565 (19)	−0.0060 (14)	0.0057 (13)	0.0112 (16)
C24	0.0410 (15)	0.0470 (17)	0.0367 (14)	−0.0058 (13)	0.0063 (12)	0.0069 (13)
C25	0.0439 (15)	0.0260 (12)	0.0311 (13)	0.0001 (11)	0.0162 (12)	0.0037 (10)
C26	0.0464 (16)	0.0259 (13)	0.0295 (13)	0.0000 (11)	0.0100 (11)	0.0012 (10)

### Geometric parameters (Å, °)

**Table:**

Cd—N1	2.326 (2)	C5—C6	1.400 (4)
Cd—N2	2.324 (2)	C6—C7	1.379 (4)
Cd—O1	2.215 (2)	C6—H6	0.9300
Cd—O5	2.406 (2)	C7—H7	0.9300
Cd—O6^i^	2.2215 (19)	C8—C9	1.477 (3)
Cd—O9	2.361 (2)	C9—C14	1.394 (4)
N1—C15	1.331 (4)	C9—C10	1.406 (4)
N1—C26	1.348 (4)	C10—C11	1.380 (4)
N2—C24	1.324 (3)	C11—C12	1.388 (4)
N2—C25	1.360 (3)	C11—H11	0.9300
O1—C1	1.256 (3)	C12—C13	1.390 (4)
O2—C1	1.258 (4)	C13—C14	1.378 (4)
O3—C3	1.366 (3)	C13—H13	0.9300
O3—H3A	0.8200	C14—H14	0.9300
O3'—C7	1.370 (18)	C15—C16	1.384 (4)
O3'—H3B	0.8200	C15—H15	0.9300
O4—C5	1.354 (3)	C16—C17	1.373 (5)
O4—H4A	0.8200	C16—H16	0.9300
O5—C8	1.254 (3)	C17—C18	1.404 (5)
O6—C8	1.279 (3)	C17—H17	0.9300
O6—Cd^i^	2.222 (2)	C18—C26	1.410 (4)
O7—C10	1.359 (3)	C18—C19	1.425 (5)
O7—H7A	0.8200	C19—C20	1.342 (5)
O8—C12	1.355 (3)	C19—H19	0.9300
O8—H8A	0.8200	C20—C21	1.438 (4)
O9—H9A	0.860 (11)	C20—H20	0.9300
O9—H9B	0.862 (14)	C21—C25	1.395 (4)
C1—C2	1.491 (4)	C21—C22	1.407 (4)
C2—C3	1.390 (4)	C22—C23	1.356 (5)
C2—C7	1.395 (4)	C22—H22	0.9300
C3—C4	1.384 (4)	C23—C24	1.392 (4)
C3—H3	0.9300	C23—H23	0.9300
C4—C5	1.382 (4)	C24—H24	0.9300
C4—H4	0.9300	C25—C26	1.452 (4)
			
O1—Cd—O6^i^	84.81 (8)	O5—C8—O6	123.0 (2)
O1—Cd—N2	165.66 (8)	O5—C8—C9	120.7 (2)
O6^i^—Cd—N2	109.02 (8)	O6—C8—C9	116.3 (2)
O1—Cd—N1	94.34 (8)	C14—C9—C10	117.4 (2)
O6^i^—Cd—N1	172.63 (8)	C14—C9—C8	121.3 (2)
N2—Cd—N1	72.45 (8)	C10—C9—C8	121.2 (2)
O1—Cd—O9	89.40 (8)	O7—C10—C11	117.7 (2)
O6^i^—Cd—O9	87.72 (9)	O7—C10—C9	121.3 (2)
N2—Cd—O9	94.81 (8)	C11—C10—C9	121.1 (2)
N1—Cd—O9	84.96 (9)	C10—C11—C12	120.0 (3)
O1—Cd—O5	83.61 (8)	C10—C11—H11	120.0
O6^i^—Cd—O5	93.93 (7)	C12—C11—H11	120.0
N2—Cd—O5	91.45 (7)	O8—C12—C11	116.7 (3)
N1—Cd—O5	93.25 (7)	O8—C12—C13	123.1 (3)
O9—Cd—O5	172.64 (7)	C11—C12—C13	120.1 (2)
C15—N1—C26	119.1 (2)	C14—C13—C12	119.2 (2)
C15—N1—Cd	125.4 (2)	C14—C13—H13	120.4
C26—N1—Cd	115.42 (17)	C12—C13—H13	120.4
C24—N2—C25	118.2 (2)	C13—C14—C9	122.2 (2)
C24—N2—Cd	126.73 (18)	C13—C14—H14	118.9
C25—N2—Cd	114.91 (17)	C9—C14—H14	118.9
C1—O1—Cd	130.9 (2)	N1—C15—C16	122.8 (3)
C3—O3—H3A	109.5	N1—C15—H15	118.6
C7—O3'—H3B	109.5	C16—C15—H15	118.6
C5—O4—H4A	109.5	C17—C16—C15	118.9 (3)
C8—O5—Cd	114.07 (16)	C17—C16—H16	120.6
C8—O6—Cd^i^	138.08 (18)	C15—C16—H16	120.6
C10—O7—H7A	109.5	C16—C17—C18	119.9 (3)
C12—O8—H8A	109.5	C16—C17—H17	120.0
Cd—O9—H9A	106 (3)	C18—C17—H17	120.0
Cd—O9—H9B	139 (3)	C17—C18—C26	117.3 (3)
H9A—O9—H9B	115 (4)	C17—C18—C19	123.0 (3)
O1—C1—O2	124.3 (3)	C26—C18—C19	119.6 (3)
O1—C1—C2	117.0 (3)	C20—C19—C18	120.8 (3)
O2—C1—C2	118.7 (3)	C20—C19—H19	119.6
C3—C2—C7	117.9 (2)	C18—C19—H19	119.6
C3—C2—C1	121.5 (3)	C19—C20—C21	121.5 (3)
C7—C2—C1	120.7 (2)	C19—C20—H20	119.2
O3—C3—C4	118.0 (2)	C21—C20—H20	119.2
O3—C3—C2	120.7 (2)	C25—C21—C22	117.7 (3)
C4—C3—C2	121.3 (2)	C25—C21—C20	119.5 (3)
C4—C3—H3	119.3	C22—C21—C20	122.7 (3)
C2—C3—H3	119.3	C23—C22—C21	119.5 (3)
C5—C4—C3	119.7 (2)	C23—C22—H22	120.2
C5—C4—H4	120.1	C21—C22—H22	120.2
C3—C4—H4	120.1	C22—C23—C24	119.2 (3)
O4—C5—C4	123.1 (2)	C22—C23—H23	120.4
O4—C5—C6	116.5 (2)	C24—C23—H23	120.4
C4—C5—C6	120.4 (2)	N2—C24—C23	123.0 (3)
C7—C6—C5	118.7 (2)	N2—C24—H24	118.5
C7—C6—H6	120.6	C23—C24—H24	118.5
C5—C6—H6	120.6	N2—C25—C21	122.2 (3)
O3'—C7—C6	123.2 (8)	N2—C25—C26	118.7 (2)
O3'—C7—C2	113.5 (8)	C21—C25—C26	119.1 (2)
C6—C7—C2	122.0 (2)	N1—C26—C18	122.0 (3)
C6—C7—H7	119.0	N1—C26—C25	118.5 (2)
C2—C7—H7	119.0	C18—C26—C25	119.5 (3)

Symmetry codes: (i) −*x*+1, −*y*+1, −*z*+1.

### Hydrogen-bond geometry (Å, °)

**Table:**

*D*—H···*A*	*D*—H	H···*A*	*D*···*A*	*D*—H···*A*
O3—H3A···O2	0.82	1.81	2.540 (3)	148
O3'—H3B···O1	0.82	1.56	2.332 (19)	156
O4—H4A···O7^ii^	0.82	1.91	2.719 (3)	172
O7—H7A···O6	0.82	1.80	2.523 (3)	147
O8—H8A···O3^iii^	0.82	1.94	2.752 (3)	169
O9—H9A···O2	0.86 (1)	1.97 (2)	2.738 (3)	148 (3)
O9—H9B···O5^iv^	0.86 (1)	2.03 (2)	2.842 (3)	158 (3)
C24—H24···O5^i^	0.93	2.58	3.433 (4)	152

Symmetry codes: (ii) *x*−1, *y*, *z*; (iii) −*x*+1/2, *y*−1/2, −*z*+1/2; (iv) *x*, *y*+1, *z*; (i) −*x*+1, −*y*+1, −*z*+1.

## References

[bb1] Altomare, A., Cascarano, G., Giacovazzo, C. & Guagliardi, A. (1993). *J. Appl. Cryst.* **26**, 343–350.

[bb2] Deisenhofer, J. & Michel, H. (1989). *EMBO J.* **8**, 2149–2170. 10.1002/j.1460-2075.1989.tb08338.x PMC401143 2676514

[bb3] Farrugia, L. J. (1997). *J. Appl. Cryst.* **30**, 565.

[bb4] Farrugia, L. J. (1999). *J. Appl. Cryst.* **32**, 837–838.

[bb5] Higashi, T. (1995). *ABSCOR* Rigaku Corporation, Tokyo, Japan.

[bb6] Li, H., Yin, K.-L. & Xu, D.-J. (2005). *Acta Cryst.* C**61**, m19–m21. 10.1107/S0108270104029580 15640568

[bb7] Rigaku (1998). *PROCESS-AUTO* Rigaku Corporation, Tokyo, Japan.

[bb8] Rigaku/MSC (2002). *CrystalStructure* Rigaku/MSC, The Woodlands, Texas, USA.

[bb9] Sheldrick, G. M. (2008). *Acta Cryst.* A**64**, 112–122. 10.1107/S0108767307043930 18156677

[bb10] Su, J.-R., Gu, J.-M. & Xu, D.-J. (2005). *Acta Cryst.* E**61**, m1033–m1035.

[bb11] Yang, Q., Zhang, L. & Xu, D.-J. (2006). *Acta Cryst.* E**62**, m2678–m2680.

